# Autoimmunity in psoriatic arthritis: pathophysiological and clinical aspects

**DOI:** 10.3906/sag-2011-235

**Published:** 2021-08-30

**Authors:** Hakan EMMUNGİL, Ufuk İLGEN, Rafi Haner DİRESKENELİ

**Affiliations:** 1 Division of Rheumatology, Department of Rheumatology, Trakya University Medical Faculty, Edirne Turkey; 2 Division of Rheumatology, Department of Rheumatology, Marmara University Medical Faculty, İstanbul Turkey

**Keywords:** Autoantibody, autoimmunity, genetic, psoriasis, psoriatic arthritis

## Abstract

Psoriatic arthritis (PsA) is an underdiagnosed entity with a broad impact on the quality of life. Although the pathogenesis is largely unknown, autoimmune footprints of the inflammation in PsA have increasingly been recognized. Most of the genetic variation predisposing to PsA is mapped to the class I major histocompatibility complex (MHC) region and shared by a variety of autoimmune diseases. Polymorphisms in the genes *IL12B*, *IL23R*, *IL13*, *TNIP1*, *TRAF3IP2*, *TYK2*, and many others explain the non-HLA genetic risk with little known functional consequences. Entheseal and synovial cellular infiltrate with oligoclonal CD8^+^ T cells and occasional germinal centers, loss of regulatory T cell function, and specific autoantibodies such as anti-PsA peptide, anti-LL-37, and anti-ADAMTSL5 are the immunopathological findings suggestive of autoimmunity. These were supported by clinical observations of autoimmune multimorbidity and treatment response to calcineurin/mTOR and co-stimulation inhibition.

## 1. Introduction

Psoriatic arthritis (PsA) is a chronic inflammatory disease that usually affects the joints, tendons, ligaments and their attachment sites, the entheses. According to a systematic review of the epidemiological studies, the prevalence of PsA ranged between 6% to 24% in patients with psoriasis when validated criteria such as Moll and Wright, classification of psoriatic arthritis (CASPAR), or European spondylarthropathy study group criteria (ESSG) were applied [1]. When the Psoriasis and Arthritis Questionnairre (PAQ) was applied, up to half of the patients defined active or past inflammatory manifestations [2]. Despite the high prevalence, PsA is still an underdiagnosed clinical entity in daily clinical practice such that undiagnosed cases constitute the majority [3]. The impact of PsA appears to be very broad and covers all aspects of life [4–6].

Psoriasis is occasionally accompanied by inflammatory conditions other than arthritis such as uveitis and inflammatory bowel disease. The concept of “psoriatic disease” was introduced to encompass the inflammatory involvement of several different organs/systems in the same patient [7]. A common genetic background with environmental triggers was proposed to underlie the inflammatory changes in different organs. Although the inflammatory manifestations such as arthritis and bowel disease share common histopathological features and cytokine profiles with the skin disease, the genetic susceptibility to psoriasis and PsA do not totally overlap (Figure) [8–17]. Since almost all patients with PsA in whom genetic susceptibility was investigated had concurrent psoriasis, most genes identified were relevant to psoriasis susceptiblity as well [9]. However, certain genes seem to be more frequently associated with PsA than psoriasis alone (i.e. the PsA-weighted genes such as *HLA-B*, *FBXL19*, and NOS2) (Figure) [8–11,13–17]. With these observations, the management of psoriasis as a systemic disease has gained attention over time [7,18]. Moreover, cardiovascular, metabolic, and psychological disorders in patients with psoriasis/PsA has been proposed to be included under a broader umbrella term “systemic psoriatic disease” [19]. Although not directly caused by an apparent or intense inflammation, diabetes, obesity, metabolic syndrome, fatty liver disease, and atherosclerosis were speculated to be at least contributed by the systemic inflammation in psoriasis/PsA [18,19]. The potential reversal of these conditions with the advancement of systemic antiinflammatory treatment modalities is an area of active research.

**Figure 1:**
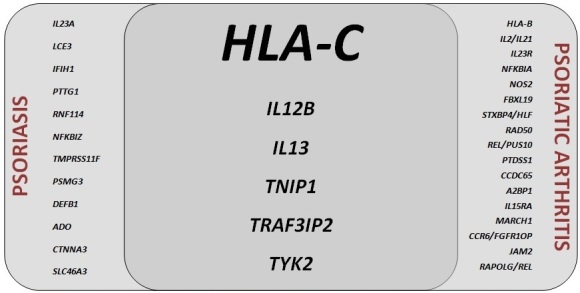
Genes implicated in susceptibility to psoriasis and psoriatic arthritis.

PsA, like psoriasis, is considered as an immune-mediated inflammatory disease with autoimmune and (auto)inflammatory features [19,20]. Spondyloarthropathies including PsA do not generally display the prototypical genetic, clinical, and immunological T- and/or B-cell mediated autoimmune diseases and T- and B-cell targeted therapies are usually not effective. Recurrent attacks, interattack remissions, local triggers, and prominently neutrophilic inflammation have made most clinicians consider spondyloarthropathies as diseases of autoinflammatory rather than autoimmune origin [21]. However, PsA is, perhaps, the “most autoimmune” disease among spondyloarthritides. Autoimmune footprints of the inflammation and autoimmune multimorbidity in PsA, which are the main topics that will be covered in this review, have increasingly been recognized.

## 2. Pathophysiological aspects

### 2.1. Genetic and epigenetic findings of autoimmunity in PsA

Familial clustering of PsA has long been known [9,22,23]. The heritability of PsA is even greater than psoriasis [23]. Most of the germline genetic risk identified so far in PsA is related to genes involved in the regulation of the adaptive immune responses such as antigen presentation and lymphocyte survival, function, differentiation, and maturation [9]. Dysregulation of the adaptive immune responses solely, of course, does not make a disease “autoimmune” but it may be speculated to at least contribute to the systemic inflammation and predispose to autoimmunity particularly if it is considered that similar genetic changes were shared by many autoimmune diseases (see below). Polymorphisms in the genes involved in the regulation of the innate immune responses such as type I interferon (IFN) signaling, nuclear factor kappa B (NF-κB) activation, and nitric oxide production also contribute to the genetic risk of PsA [8,9]. Innate immune dysregulation has been known to modify the course of autoimmune diseases [24]. Indeed, monogenic forms of systemic lupus erythematosus (SLE), a prototype systemic autoimmune disease, are mostly caused by mutations in the genes involved in the regulation of the innate immune responses such as nucleic acid sensing and degradation and type I IFN signaling [25]. Major gene variants implicated in PsA will be discussed below along with functional consequences and possible autoimmunity-related features. It should be noted that all genome-wide association studies (GWAS) in PsA were performed only in European ancestry cohorts [9,13,14]. Ethnic charactersitics of the cohorts were of importance in previous linkage studies in PsA [26] and GWAS in psoriasis [27]. Another important point is that there is a paucity of studies that report the functional consequences of the genetic changes in PsA which makes the interpretation of the proposed associations difficult. Although GWAS have identified a significant proportion of the genetic risk to PsA over the last decade, the biological translation of these findings is not straightforward. GWAS typically assign single-nucleotide polymorphisms to the nearest gene but most of the time many variants simultaneously exist with high linkage disequilibrium. Despite advanced bioanalytical methods such as functional enrichment, network-assisted and pathway analyses, less than a half of the genetic variation could be identified to be truely functionally relevant in immune cells making functional studies imperative to understand the disease pathogenesis and guide treatment strategies [28]. Functional studies even may fail to determine the consequence(s) of a variation if issues such as “cells or tissues of interest”, “stimulated or resting state”, and “healthy or diseased subjects” are not properly addressed [28].

#### 2.1.1. Human leukocyte antigen (HLA) associations

After the identification of PSORiasis susceptibility (PSORS1) gene on 6p21.3 as the major genetic determinant of psoriasis, candidate gene and linkage studies confirmed this association in PsA as well [9–11,29]. The PSORS1 risk variant, carried by more than half and about one third of the patients with psoriasis and PsA, respectively, was later identified to be the HLA-C*06:02 (corresponding to the HLA-Cw6 serotype) [30] and other polymorphisms in the major histocompatibility complex (MHC) class I region such as HLA-B*27, -B*38, and -B*39 have repeatedly been noted in PsA but not in psoriasis [9–11,13–17,29]. According to the most recent genome-wide meta-analysis, [13] association of most of the other loci in the MHC region (including the non-*HLA* genes) with PsA seemed to be attributable to linkage disequilibrium with the HLA-B/‑C region as previously shown to be the case in psoriasis as well [31]. HLA polymorphisms are not only related to PsA risk but also the disease phenotype. HLA-B27 was found to be associated with axial disease whereas HLA-B38 and -B39 were more frequent in peripheral polyarthritis [9,10,29,32,33]. HLA-B39 was related to progressive disease as well [32,33]. Some *HLA* polymorphisms that were not associated with PsA risk independently may still modify the disease course. HLA-DR7 (HLA-DRB1*07 gene product), an MHC class II antigen, was found protective against disease progression and HLA-DQw3 (HLA-DQB1*03 gene product) was associated with progressive disease but only in the absence of HLA-DR7 [33,34]. Importantly, PsA patients carrying rheumatoid arthritis (RA) shared epitope alleles were found to have more radiological erosions although their presence was not linked to an increased PsA risk [35]. 

Given their strong risk association, explaining the functional role of the *HLA* risk alleles, particularly the HLA-C*06:02, is essential for elucidating the pathogenesis of PsA. A fascinating case study with a unique methodology identified ADAMTS-like 5 (ADAMTSL5) as an HLA-C*06:02-presented melanocytic autoantigen to the lesion-infiltrating autoreactive CD8^+^ T cells in psoriasis [36]. More interestingly, peptide motifs of the HLA-C*07:01, -C*07:02, and -B*27, which are three of the other psoriasis risk-related leukocyte antigens, utilize the same anchor residues with the HLA-C*06:02, have overlapping peptide-binding properties, and belong to the same HLA supertype [37]. Synovial/entheseal counterpart of this picture is yet to be studied but seems to be more complicated since different disease phenotypes in PsA exhibit different HLA associations and contribution of the HLA-C*06:02 polymorphism to PsA risk is lesser compared to psoriasis [38].

Class I MHC molecules are predominantly found anchored in the cell membrane as trimeric structures consisting of a heavy chain, a bound peptide, and β_2_-microglobulin. However, the heavy chains of the class I MHC molecules can dissociate from the bound peptide and β_2_-microglobulin and assume an open conformation [39]. These free heavy chains can form homodi/trimers and observed more frequently on the activated CD8^+^ T cells, and are implicated in many metabolic and immune cellular processes such as glucose uptake, cell growth, interleukin-2 (IL-2)-, and T cell receptor (TCR)-mediated signaling via *cis*-interactions with the insulin, epidermal growth factor, IL-2 receptors, and TCR, respectively [39,40]. HLA-C has a weaker association with β_2_-microglobulin than HLA-A or -B, making dissociation from β_2_-microglobulin and bound peptide more favorable [40]. Interestingly, similar is true for the HLA-B27 as well [41]. β_2_-microglobulin liberated from the disintegrated MHC is also a trigger for synoviocytes to release inflammatory mediators resulting in tissue destruction [40].

An important feature of the HLA-C is its interaction with killer immunoglobulin-like receptors (KIRs) mainly expressed on the natural killer (NK) cells. One hundred percent of the HLA-C types but only a minority of the HLA-A and -B (but including HLA-B27) subsets recognize KIRs [40]. Another consequence of the open conformation and homodi/trimerization is activation of the NK cells via altered binding to KIRs [39–41]. Inhibition of binding of HLA-B27 homodimers to KIR-3DL2 by a specific antibody to HLA-B27 homodimers resulted in inhibition of survival and proliferation of KIR-3DL2^+^ NK cells [42]. This antibody also inhibited production of IL-17 by peripheral blood mononuclear cells from patients with spondyloarthritis possibly via inhibition of binding of HLA-B27 homodimers to KIR-3DL2 expressing CD4^+^ T cells [42]. Recently, some *KIR* polymorphisms were shown to be associated with PsA risk [43] although they were not reported to be significant at the genome-wide level in GWAS meta-analyses [13,14].

*HLA-B *and *-C* associations of significantly increased genetic risk to selected autoimmune and immune mediated inflammatory diseases are summarized in Table 1 [44–64]. Many autoimmune diseases such as type I diabetes, myasthenia gravis, SLE, Sjögren’s syndrome, pemphigus vulgaris, dermato/polymyositis, and vitiligo share HLA associations with PsA, the last being associated with both HLA-C*06:02 and *-*B*27 [59,60]. It is an interesting finding since CD8^+^ T cell autoreactivity against a melanocyte antigen presented on HLA-C*06:02 was demonstrated in psoriasis [36] and peptide-binding properties of HLA-C*06:02 and -B*27 overlap [37]. Most other associations of common genetic risk to PsA and autoimmune diseases lie in the HLA-C*07:01, which is the HLA-C constituent of the the ancestral Caucasian haplotype AH8.1 (the Super B8, HLA-A*01:01-C*07:01-B*08:01-DRB1*03:01-DRB3*01:01-DQA1*05:01-DQB1*02:01) [65]. This particular haplotype and its fragments, carried as a whole in 18% and at least as one constituent in 36%–39% of the healthy Caucasian population [66] and more frequently in northern Europe, have been found to be associated with numerous autoimmune diseases and abnormal immune responses even in healthy population (the 8.1 effect) [65]. It is important to underline here again that the HLA-C*07:01, -C*06:02, and -B*27 belong to the same HLA supertype according to peptide binding properties [37].

**Table 1:** HLA-B and -C associations of the significantly increased genetic risk to selected autoimmune and immune mediated inflammatory diseases.

Disease	DominantMHC Association	HLA-B and -CAssociation	Effect size(OR with 95% CI)	Ref.
Type I Diabetes	Class II (HLA-DQ&-DR)	B*18:01	2.05 (1.59-2.61)a	44
		B*39:06	10.31 (4.21-25.1)a	44
		C*03:03	1.48 (1.10-1.99)a	44
Autoimmune Thyroid Disease				
Graves’ Disease	Class II (HLA-DQ&-DR)	B*08†	2.8 (1.81-4.33)b,c	45,46
Autoimmune Hypothyroidism	Class II (HLA-DQ&-DR)	-	-	46
Myasthenia Gravis	Class I&II (HLA-B,-C,&-DQ)	B*08:01†	5.1 (2.48-10.5)d	47
		C*07:01†	1.85 (1.14-3.01)d	47
Multiple Sclerosis	Class II (HLA-DR)	C*15	2.58 (1.57-4.25)a	48
Celiac Disease	Class II (HLA-DQ)	-	-	49
Rheumatoid Arthritis	Class II (HLA-DR)	-	-	50
Systemic Lupus Erythematosus	Class II (HLA-DR)	B*08:01†	1.84 (1.7-1.99)a	51
		C*07:01†	1.57 (1.47-1.69)a	51
Sjögren’s Syndrome	Class II (HLA-DR&-DQ)	B*08†	5.27 (2.31-12)a	52
		B*35:01	3.7 (1.92-7.12)c	53
		C*07†	5.23 (2.24-12.21)c	54
Systemic Sclerosis	Class II (HLA-DR)	-	-	55
Autoimmune Bullous Diseases				
Pemphigus Vulgaris	Class II(HLA-DR)	B*38	4.32 (2.22-8.38)c	56
		B*57	3.55 (1.64-7.65)e	57
		C*12	3.15 (1.87-5.3)c	56
Bullous Pemphigoid	Class II(HLA-DR&-DQ)	C*15	2.97 (1.45-6.1)e	57
		B*37:01	8 (3.35-19.17)f	58
		C*17	8.31 (2.46-28.16)f	58
Vitiligo	Class I&II(HLA-A,-B,-C&-DR)	B*13	1.87 (1.23-2.84)f	59,60
		B*27	2.29 (1.3-4.06)f	59
		C*06:02	3.04 (1.79-5.18)f	59,60
Autoimmune Liver Diseases				
Autoimmune Hepatitis	Class II (HLA-DR)	-	-	61
Primary Biliary Cholangitis	Class II (HLA-DR)	-	-	62
Primary Sclerosing Cholangitis	Class II (HLA-DR)	B*08†	2.99 (1.83-4.9)c	63
Idiopathic Inflammatory Myopathies				
Polymyositis & Dermatomyositis	Class I&II (HLA-A,-B,-C,-DR&-DQ)	B*08:01†	2.5 to 4.6	64
		C*07:01†	2.1 to 3.6c	64
Inclusion Body Myositis	Class I&II (HLA-A,-B,-C,-DR&-DQ)	C*14	29.3 (2.68-1449.2)c	64

Abbreviations: HLA = Human leukocyte antigen, MHC = Major histocompatibility complex, OR = Odds ratio, CI = Confidence interval.aafter accounting for linkage disequilibrium (LD) with dominant associations bprobably due to LD with HLA-DR c not tested for LD dcontrolled for each other. econtrolled for other HLA-B and -C alleles f controlled for other class I alleles tested.†the ancestral Caucasian haplotype AH8.1 (HLA-A*01:01 -C*07:01 -B*08:01 -DRB1*03:01 -DRB3*01:01 -DQA1*05:01 -DQB1*02:01) relation may be of concern.

#### 2.1.2. Non-HLA associations in the MHC region

Although MHC class I polypeptide-related sequence A (MICA), independent of the HLA*-*C*06:02, and tumor necrosis factor-α (TNFA) genes were previously identified as PsA risk regions in relatively small-scale studies, [67–70] these findings could not be replicated in different races and in large GWAS and meta-analyses [13,14], and at least partially explainable by linkage disequilibrium with the HLA-B and –C [9,10,33]. Anyway, MICA-A9 (MICA*002 gene product), expressed on various cell types including keratinocytes, fibroblasts, and synoviocytes, was speculated to act as a superantigen independent of antigen presentation on the MHC [67]. More recently, different *MICA* and *MICB* polymorphisms were implicated in immune response since the products of these genes are ligands for NKG2D (natural killer receptor group-2 D, encoded by the gene killer cell lectin like receptor K1, KLRK1), a master regulator of immune cell responsiveness primarily expressed on all NK, NKT, and some T cell subsets [71,72]. MICA/MICB-NKG2D interactions result in phosphatidylinositol-3 kinase activation ending up with many cellular processes like survival, activation, and proliferation [71,72]. While one can doubt why these innate immune response elements be important to understand the autoreactivity in PsA, the fact is that MICA and B polymorphisms are related to the genetic risk in other autoimmune diseases such as celiac disease, RA, and multiple sclerosis (MS) [73]. Experiments in RA patients and in autoimmune encephalomyelitis and collagen-induced arthritis models revealed the functional consequences of MICA/MICB-NKG2D interactions such as T cell autoreactivity, co-stimulation of TCR-mediated cytokine release, and proliferation [73]. So, as previously mentioned, innate immune dysregulation seems like more than a bystander in autoimmune disease pathogenesis.

Many polymorphisms in the TNFA, particularly in the promoter region, were reported to be related to PsA risk either dependent or independent of the HLA-C*06:02 [69,70] but with inconsistent functional results, i.e. the TNF-α mRNA and protein expression, in healthy people and disease groups including PsA [74–76] Nevertheless, the promoter polymorphisms of the TNFA are associated with autoimmune diseases including SLE and RA [76]. Indeed, overexpression of only TNF-α was sufficient enough to cause chronic severe erosive polyarthritis resembling RA in mice transfected with human TNF-α gene engineered for enhanced transcription [77].

#### 2.1.3. Genetic associations outside the MHC region

Among more than 20 genetic susceptibility loci to PsA, only few have consistent and strong associations [9,13,14] (Figure) and will be reviewed here. But it is important to underline that some polymorphisms not found to have genome-wide significant effect on PsA risk in GWAS and meta-analyses may still be players in disease pathogenesis. One example is the *KIR*, polymorphisms of which were found to be associated with PsA in case-control studies [43] but not identified as a genetic risk region in large-scale GWAS [13,14] although a functional consequence is likely since class I MHC molecules (including HLA-C*06:02 and -B*27) are ligands for these receptors [39–42] but not demonstrated in PsA or psoriasis to date. Interestingly, functionally relevant combinations of HLA and KIR genotypes may confer protection or susceptibility to PsA [78]. KIR genes were also implicated in susceptibility to RA [79].

##### 2.1.3.1. Cytokine and cytokine receptor genes: IL-12 subunit β (p40) (IL12B), IL-23 receptor (IL23R), and IL13

IL-12 subunit β (p40) is a component of both IL-12 and -23. Biological activity and receptor binding of both cytokines are conferred by the shared p40 subunit [80]. Although variation within IL12B and IL23R was shown to be associated with PsA independent of both each other and the MHC region [9,13,14], the functional consequences of the implicated polymorphisms have not been demonstrated clearly. Given the strong evidence of IL-12 and IL-23/IL-17 axis driven inflammation in PsA, the implicated polymorphisms were hypothesized to alter the expression and/or function of these molecules. This was further supported by the association of PsA risk with rs321227 and rs2201841, which are located within the 3’-untranslated (possibly affecting transcription) and intergenic (possibly affecting splicing) regions of the IL12B and IL23R, respectively [81,82]. rs11209026, located in the Janus kinase 2 (JAK-2)-binding domain of the IL23R*,* was found to be associated with PsA risk in at least three different studies [9,81,82] and healthy people carrying the low-risk minor variant (R381Q) were found to have lower numbers of circulating Th17 and Tc17 cells compared to those carrying the wild-type variant [83]. More importantly, CD4^+^ and CD8^+ ^T cells from these individuals show decreased IL-23-mediated Th/c17 cytokine production and expansion [83]. Moreover, p40 subunit deficient mice were protected from developing collagen-induced autoimmune arthritis, experimental autoimmune antiglomerular basement membrane disease, and experimental autoimmune encephalomyelitis [80] highlighting the role of IL-23 in autoimmunity. Recently, certain IL12B and IL23R polymorphisms were found to be associated with alopecia areata in man [84] but they were previously reported not to be associated with RA and SLE [85,86].

*IL13* polymorphisms were proposed as PsA markers among psoriasis patients in the earlier reports [87,88] but this gene was later identified as a risk region for cutaneous-only psoriasis as well albeit with a weaker association in a large GWAS meta-analysis [13]. IL-13 is a Th2 cytokine produced during allergic inflammation. It mediates the survival of eosinophils, activation of fibroblasts (leading to fibrosis), and immunoglobulin M (IgM)-to-IgE class-switch, and genetic polymorphisms in the *IL13* were mostly associated with asthma, allergic rhinitis, and atopic dermatitis ClinVar (2021). Genomic variation and its relationship to human health [online]. Website https://www.ncbi.nlm.nih.gov/clinvar [accessed 19 March 2021]. [87,88]. Although PsA patients were known to have higher IL-13 levels in their synovial fluids (but not sera) compared to patients with RA and osteoarthritis, [89] the role of *IL13* polymorphisms on that finding and in the pathogenesis of PsA and autoimmune diseases remains to be elucidated since IL-13 is generally considered as an inhibitory regulator of Th1- and Th17-mediated inflammation [90]. IL13 polymorphisms were not found to be associated with genetic risk to type I diabetes, RA, systemic sclerosis (SSc), and Sjögren’s syndrome although serum IL-13 levels were mostly elevated [91]. Exceptionally, minor variant of the rs20541, previously implicated in PsA, [87,88] was shown to be associated with SLE and renal involvement in a case-control study from China with significantly increased IL-13 levels in the carriers [91]. But the result could not be replicated in an SLE GWAS meta-analysis [92].

##### 2.1.3.2. TNF-α-ınduced protein 3-interacting protein 1 (TNIP1) and TNF receptor associated factor 3-interacting protein 2 (TRAF3IP2) genes

*TNIP1* encodes a TNF-α-induced protein 3 (TNFAIP3 or A20)-binding protein which plays a role in autoimmunity and tissue homeostasis through the regulation (repression) of NF-κB activation downstream of TNF-α and Toll-like receptors. Polymorphisms in this gene have been associated with psoriasis, PsA, RA, SLE, and SSc [93,94]. TNIP1 has also been shown to function in signal repression even in the absence of TNFAIP3 [94,95]. Like TNIP1, TNFAIP3 was identified as a genetic risk region for psoriasis and PsA [9]. Notably, pathogenic mutations in TNFAIP3 cause an autoinflammatory disease with autoimmune features, haploinsufficiency of A20, in humans [95]. Experimental overexpression of TNIP1 decreased cells’ responsiveness to irritants and prevented an active state in vitro and a lupus-like autoimmune phenotype was observed in mutant *TNIP1* knock-in mice but interestingly not in TNIP1 knock-out ones [94]. SLE, SSc, RA, and proriasis patients carrying the variants of this gene were shown to have altered mRNA and protein expression [93,94]. Possible functional impacts of disease-related polymorphisms and their exact role in the pathogenesis were not reported to be studied in PsA.

TRAF3IP2 (Act1) was first identified as an ınhibitor of NF-κB kinase (IKK)-binding protein and experimental disruption of the IKK-binding domain of this protein abolished NF-κB activation. Interestingly, it associates with IL-17 receptor as well and expression of IL-17-dependent inflammation-related genes was abolished in Act1-deficient mouse cells. Binding of IL-17A/F to IL-17 receptor leads to recruitment of Act1 and this allows TNF-receptor associated factor 6 (TRAF6) binding to Act1. Act1-TRAF6 signaling complex, then, mediates the downstream activation of both NF-κB and mitogen activated protein kinase (MAPK) pathways [96]. Act1 is also a negative regulator of CD40- and B cell activating factor belonging to the TNF family (BAFF)-mediated B cell survival [97]. Mice lacking TRAF3IP2 develop Sjögren’s syndrome in association with lupus nephritis due to hyper-B cell function but contrarily they show delayed and less severe experimental autoimmune encephalomyelitis which was thought to be IL-17 mediated [97]. TRAF6-binding site of Act1 was needed to respond to IL-17 (but not to TNF-α) and a polymorphism, rs33980500(C→T), nearby this site was shown to be associated with psoriasis and PsA Online Mendelian Inheritance in Man® (OMIM®) (2021). An Online Catalog of Human Genes and Genetic Disorders [online]. Website https://www.omim.org [accessed 19 March 2021].) [9,98]. Functional assays showed reduced binding of this TRAF3IP2 variant to TRAF6 in PsA [98]. Missense mutations in *TRAF3IP2* in humans are associated with chronic familial mucocutaneous candidiasis but not with autoimmune disease^1^.

##### 2.1.3.3. Nonreceptor tyrosine kinase 2 gene (TYK2)

TYK-2 plays an indispensable role in controlling responses to multiple cytokines in humans. Interferon receptor (IFNAR1) expression on the cell surface and signaling through IL-6, -10, -12, and -23 receptors are dependent on an intact TYK-2, which is involved in the activation (by tyrosine phosphorylation) of multiple Janus kinase/signal transducer and activator of transcription (JAK/STAT) pathways in human T cells^3^. Pathogenic mutations of the TYK-2 results in an immunodeficiency syndrome, the Immunodeficiency-35, in man^3^. Polymorphisms of this gene were found to be associated with a variety of immune mediated inflammatory and autoimmune diseases including psoriasis and PsA, MS, SLE, RA, type I diabetes, idiopathic inflammatory myopathy, SSc, primary biliary cholangitis, inflammatory bowel disease, and ankylosing spondylitis [99]. While most minor alleles are protective, the same polymorphic variant may be protective for one disease but risky for another one [99]. Functional consequences of many of these variants were studied in vitro in human cell lines and in healthy and diseased subjects. Splicing, mRNA and protein expression, enzymatic activity, and downstream signaling were demonstrated to be altered for some variants in different human diseases [99]. Peripheral blood mononuclear cells and skin-homing CD4^+^ and CD8^+^ lymphocytes from psoriasis patients carrying the protective variant (rs12720356 encoding I684S) manifested reduced phospho-STAT4 levels upon stimulation compared to those carrying the wild-type and risk-associated variant (rs34536443 encoding P1104A) [100]. No study reported the functional consequences of *TYK2* variants in PsA.

#### 2.1.4. Epigenetics and autoimmunity in PsA

Early studies identified a parent-of-origin effect on the heritability of psoriasis and PsA (i.e. a more prominent paternal transmission) [101]. This finding was later linked to genomic imprinting, an epigenetic phenomenon. Subsequent studies identified that some genetic associations of PsA such as nucleotide-binding oligomerization domain-containing protein 2 (NOD2) and HLA-B*08 were more specific to paternally transmitted disease [101]. Candidate gene and genome-wide methylation, histone modification, regulatory RNA, and chromatin remodelling studies followed these but unfortunately only in psoriasis. Studies on the epigenetic mechanisms of disease are scarce in PsA. A search with the keyword “psoriatic arthritis” yielded only a total of six results in the five PubMed/MEDLINE-indexed epigenetics journals (Clinical Epigenetics, Environmental Epigenetics, Epigenetics, Epigenomics, and Epigenetics & Chromatin in alphabetical order) as of November 2020 none being a PsA study. Similarly, search terms “psoriatic arthritis” and “epigenetics” yielded 26 results in the entire PubMed, 19 of which being reviews regarding psoriatic disease, with only two epigenetic studies in PsA.

Global hypomethylation is a common feature of many autoimmune and immune mediated inflammatory diseases including PsA [101] and is proposed to lead to enhanced gene expression and hyperactivity in T and B cells [102]. However, differential hypo/hypermethylation and histone modifications such as acetylation, phosphorylation, and ubiquitination of the particular genes (leading to altered transcription) involved in immune functions and microRNA-mediated post-transcriptional regulation of expression are of concern. According to a pilot epigenome-wide sperm/blood methylation study in psoriasis and PsA, differentially methylated regions were found to be enriched within the MHC but not in close proximity to the class I gene loci [103]. Whether these represent distal regulatory elements remains to be determined but top differentially methylated regions contained potential biologically relevant genes including HLA complex group *26* (HCG26*,* which lies adjacent to the HLA complex *P5* and between the MICA and MICB, all of which are associated with genetic risk to PsA and autoimmune diseases including SLE, RA, and type I diabetes) and Integrin Subunit β2 (*ITGB2*) in PsA probands [103]. Although the effect of hypo/hypermethylation of a gene region is predictable in terms of expression, it still needs confirmatory functional studies since an epivariation is only a small point in a huge complex genetic background of PsA. Nevertheless, variations in the *HCG26* and *ITGB2* were previously reported to be associated with MS, type I diabetes, and RA [104]^1^. 

Peripheral blood microRNA profiles of patients with active PsA were found to be different compared to patients with inactive disease and healthy controls [105]. Pathway and interaction network analyses revealed some differentially expressed microRNAs to be involved in TNF, MAPK, and WNT signaling exclusively in active PsA. miR-126-3p, highly downregulated in active PsA, was shown to target and inhibit the expression of genes involved in immune functions including phosphoinositide-3-kinase regulatory subunit 2 (PI3KR2) and receptor activator of NF-κB şigand (RANKL) in transfected human T cell leukemia cell line [105]. Both miR-126-3p [106] and several other dysregulated microRNAs in PsA were reported to be of pathogenetic and even potential diagnostic importance in autoimmune diseases including RA, SLE, MS, and dermatomyositis [106,107].

### 2.2. Immunopathological findings of autoimmunity in PsA

Genetic predisposition and triggers such as dysbiosis, mechanical stress and injury, obesity, and infections were proposed to cause aberrant immune cell activation and secretion of inflammatory cytokines leading to inflammation in the skin, synovium, entheses, tendons, periosteum, bone, and gut [108]. Key effector cells and cytokines change according to the tissue involved and chronology of the disease. Th1 and Th/c17-mediated inflammation is the hallmark of PsA with the key cytokines TNF-α, IL-1β, -9, 23, -17, and -22 [108]. Although these cells and cytokines have pathogenetic impacts in any autoimmune disease, findings of clear autoreactivity presumable to reflect immunity to self (neo)antigens and loss of tolerance will be reviewed here.

#### 2.2.1. Potentially autoreactive T cells in PsA

The cellular infiltrate in the psoriatic skin, synovium, and inflammatory enthesis is similar and predominantly lymphocytic [109]. Although the T cells (mainly CD8^+^) are predominant in the synovial fluid and entheseal inflammation, B cells may occasionally form germinal centers (i.e. ectopic lymphoid neogenesis) in the affected synovia [109]. A subset of synovial fluid and synovial CD8^+^ T cells show TCR oligoclonality suggesting an antigen driven response in PsA [109,110]. Although no autoantigen could be identified for these (oligo)clonal CD8^+^ T cells with highly homologous complementarity-determining regions, they are present in the effector memory cell phenotype in the systemic circulation as well [111]. Upon stimulation, IL-17and IFN-γ/IL-17 double producing T cells were expanded in this peripheral CD8^+^ T cell subset with ability to migrate to synovial fluid.[111] Interestingly, CD4^+^Foxp3^+^ regulatory T (Treg) cells were shown to be induced to secrete IL-17 and lose suppressor activity (exTreg cells) in the inflamed tissue microenvironment in PsA [112]. This may lead to loss of immune tolerance. The role of antigen presenting cells such as dendritic cells and macrophages in the context of autoimmunity remains to be elucidated in the pathogenesis of PsA.

#### 2.2.2. Autoantibodies in PsA

Although autoantibodies such as rheumatoid factor (RF), anticitrullinated peptide (ACPA), antinuclear (ANA), and antineutrophilic cytoplasmic (ANCA) antibodies may occasionally be encountered in PsA patients’ sera, their role in disease pathogenesis is not clear but ACPA, being present in only 0.9% to 17.5%, was found to be associated with polyarticular and erosive disease [113]. ANA prevalence was similar or slightly increased compared to that of general population if patients on biologics were excluded [113,114]. Other autoantibodies implicated in RA such as antimutated citrullinated vimentin (anti-MCV) and anticarbamylated peptide (anti-CarP) were reported to be present in up to one third of the PsA patients and proposed to be used as diagnostic/prognostic disease markers [113,114]. Beyond these shared autoantibodies, more specific ones with possible roles in disease pathogenesis were identified. Anti-PsA peptide antibody was detected in the sera of 85% of the PsA and 8% of the RA patients while not present in healthy controls’ sera [115]. It was present only in 3.3% patients with psoriasis and not detected in the sera of patients with SLE, Sjögren’s syndrome, SSc, and ankylosing spondylitis [115]. This PsA peptide (TNRRGRGSPGAL dodecamer) exhibited sequence homology with cutaneous (fibrillin-3, desmocollin-3, and keratin-78) and entheseal antigens (nebulin-related anchoring protein, N-RAP) and with IL-17B and toll-like receptor 2 (TLR2) [115]. PsA patients’ sera cross-reacted with these cutaneous and entheseal autoantigens and TLR2 [115]. Two antigens expressed in the psoriatic skin, ADAMTSL5 and a cathelicidin, LL-37, towards which an autoimmune activity was shown to be directed in psoriasis, were identified as autoantigens in PsA as well [116]. IgG autoantibodies against ADAMTSL5 and LL-37 were shown to be present in PsA even with higher concentrations than in psoriasis-only patient sera significant enough to differentiate PsA and psoriasis [116]. Interestingly, SLE but not atopic dermatitis controls were reported to have anti-ADAMTSL5 and anti-LL-37 IgG serum levels similar to those of psoriasis patients [116]. Neutrophil extracellular trap (NET)-derived DNA-LL-37 complex was shown to be able to activate B cells leading to anti-LL-37/anti-NET autoantibody production in SLE [117]. Although implicated as a potential source of autoantibodies in psoriasis, [118] NETosis was not studied in PsA. More than 40 different antibodies (eight of which were present in more than half of the patients with 2.86 to 10.59 times higher serum levels compared to healthy people) to autoantigens have been identified in psoriasis to date [116] and most remain to be studied in PsA.

## 3. Clinical observations

Given the shared genetic risk factors and immunopathological mechanisms, PsA patients may be expected to have more frequent autoimmune multimorbidity. Although controlled studies are lacking, autoimmune diseases such as SLE, celiac disease, vitiligo, and thyroid autoimmunity seem to be more frequent in PsA than that expected in the population (Table 2) [119–125]. These observations should be confirmed in large PsA cohorts. The opposite may also be true but difficult to demonstrate particularly if the autoimmune disease itself is arthritic. If one considers a research on the frequency of RA in patients with psoriatic disease, the diagnoses will be equivocal in many cases. Similarly, it is quite difficult to conduct a research on the prevalence of psoriatic disease in RA. Nevertheless, psoriasis was reported to be more frequent than expected in large SLE and SSc cohorts and in MS although no PsA data were provided [126–128].

**Table 2:** The prevalence of selected autoimmune diseases in psoriatic arthritis.

Autoimmune Disease	PrevalencePsoriatic Arthritis	PrevalenceControl Group	Ref.
Thyroid Autoimmunity*	33/97 (34%)	15/97 (15.5%)	121
	F:12/36 (33.3%)	F:33/180 (18.3%)	122
	M:11/44 (25%)	M:10/220 (4.5%)	122
Systemic Lupus Erythematosus	18/4836 (0.37%)‡	36/24180 (0.15%)	123
Sjögren’s Syndrome	1/41 (2.4%)	-	124
Celiac Disease	11/3161 (0.35%)	74/31610 (0.23%)	125
	5/114 (4.4%)†	-	126
Idiopathic Inflammatory Myopathies			127
Polymyositis & Dermatomyositis	2/1228 (0.16%)‡	-	
Inclusion Body Myositis	2/1228 (0.16%)	-	
Vitiligo	3/114 (2.6%)†	-	126
Alopecia Areata	1/114 (0.9%)	-	126
Atrophic Gastritis	1/114 (0.9%)	-	126

Abbreviations: F = Female, M = Male.*with or without thyroid dysfunction †higher than the expected prevalence in the population. ‡

Lastly, treatment response to co-stimulation inhibiton and calcineurin/mammalian target of rapamycin (mTOR) inhibiton may suggest contribution of autoimmunity to inflammation in PsA. B-cell depletion with rituximab may have some efficacy in PsA patients with long-standing disease [129]. Pleitropic drugs such as corticosteroids and conventional disease-modifying antirheumatic drugs commonly used in the treatment of PsA are effective in systemic autoimmune diseases as well. Clinical trials of biological drugs targeting IL-23 and IL-17, which are the cornerstones in treatment of psoriatic disease, are under way with preliminary success in autoimmune diseases such as SLE and MS [130,131].

## 4. Conclusion

The pathogenesis of the PsA is largely unknown. But there is some evidence of autoimmunity in PsA as suggested by genetic changes shared by a variety of autoimmune and immune mediated inflammatory diseases, immunopathological findings such as synovial oligoclonal CD8^+^ T cells and circulating autoantibodies, and clinical observations of autoimmune multimorbidity.
